# 

**Published:** 1827-10

**Authors:** 


### MONTHLY LIST OF MEDICAL BOOKS.

An Introduction to the Comparative Anatomy of Animals; by C. G. CARUS, M.D. &c. Translated from the German by R. T. GORE, M.R.S. 2 vols. 8vo. and a 4to. vol. of twenty Plates, with explanatory References.

Manual of Pathology; by L. MARTINET, D.M.P. Translated, with Notes and Additions, by JONES QUAIN, A.B.—18mo. 297.—London, 1827.

A Practical Treatise on the Diseases of the Skin. Second Edition, corrected and enlarged. By S. PLUMBE, M.R.C.S. &c. &c.—8vo. pp. 470.

Introduction to the Science of the Pulse, as applied to the Practice of Medicine. By JULIUS RUCCO, M.D. Bachelor of Medicine in the Royal University of Naples; ex-Professor of Anatomy and Comparative Physiology in the Royal Medico-Chirurgical College, &c. &c. &c. Vol. I.—8vo. pp 353.

An Essay on Morbid Sensibility of the Stomach and Bowels, as the Proximate Cause, or Characteristic Condition of Indigestion, Nervous Irritability, Mental Despondency, Hypochondriasis, &c. &c. By JAMES JOHNSON, M.D. of the Royal College of Physicians, and Physician to H. R. H. the Duke of Clarence, &c. Fourth Edition.—8vo. pp. 157. London, 1827.

Medical Botany, No. VIII.; containing Viola Odorata, Cassia Senna, Papaver Rhæas, and Acorus Calamus.

————————, No. IX.; containing Gratiola Officinalis, Momordica Elaterium, Œnanthe Crocata, and Geum Urbanum.

*** We have again to repeat the high encomiums we have more than once bestowed on the manner in which this work is executed.

*The Papers of* Mr. GUTHRIE, Dr. VARLEZ, Mr. NORTH, Mr. HAINWORTH, *and* Dr. GREGORY, *have been received.*

